# Barriers and Facilitators in Rehabilitation in Chronic Diseases and After Surgery: Is It a Matter of Adherence?

**DOI:** 10.7759/cureus.20173

**Published:** 2021-12-05

**Authors:** Elijah E Sanches, Emily Aupers, Nasser Sakran, James Navalta, Tomasz Kostka, Sjaak Pouwels

**Affiliations:** 1 Surgery, Leiden University Medical Center, Leiden, NLD; 2 Surgery, Haaglanden Medical Center, The Hague, NLD; 3 Intensive Care Medicine, Elisabeth-Tweesteden Hospital, Tilburg, NLD; 4 Surgery, Emek Medical Center, Afula, ISR; 5 Kinesiology and Nutrition Sciences, University of Nevada Las Vegas School of Medicine, Las Vegas, USA; 6 Geriatrics and Sports Medicine, Medical University of Lodz, Lodz, POL

**Keywords:** pulmonary rehabilitation, cardiac rehabilitation, complications, surgery, preoperative exercise therapy

## Abstract

Although it has been generally acknowledged that participating in rehabilitation programs is better for chronic diseases or post-surgery, the adherence rates of these programs remain lower than expected. According to the World Health Organization (WHO), adherence has been defined as follows: "the extent to which a person's behavior corresponds with agreed recommendations from a healthcare provider." In general, rehabilitation is well investigated, and in chronic diseases like chronic obstructive pulmonary disease (COPD), cardiovascular disease, neuromuscular disease, cancer, and even psychiatric diseases like depression, it has been shown that exercise therapy, in particular, has beneficial effects on morbidity, mortality, and convalescence of these patients. The aim of this review is to give an overview of the barriers and facilitators in rehabilitation practices and possible reasons why adherence rates remain low. Regarding potential future research, barriers and facilitators also need to be taken into account. Despite promising research in the field of preoperative exercise therapy (PET) and preoperative rehabilitation (prehab) and the enormous body of evidence in postoperative rehabilitation or rehabilitation in chronic diseases, it is also needed to take into account the accessibility of these prehab facilities in research and in clinical practice.

## Introduction and background

Although participation in rehabilitation programs has shown effectiveness in patient outcomes in chronic diseases and postoperative care, adherence rates remain lower than expected [1]. According to the World Health Organization (WHO) "adherence" has been defined as follows: “the extent to which a person's behavior corresponds with agreed recommendations from a healthcare provider” [2,3]. Rehabilitation is a person-centered health strategy, including exercise, safety measurements, and aiding devices, to reduce the impact of certain health conditions or surgery [1-4].

It is well known that, for example, cardiac rehabilitation reduces the risks of myocardial infarction, hospitalization, and death in patients with chronic heart failure [1]. Cardiac rehabilitation consists of multiple aspects of which supervised exercise and lifestyle education are the most important. It also involves education related to a healthy lifestyle and psychoeducation if needed. Most cardiovascular societies in the United States are endorsing these programs, and it is often (partly) reimbursed by the insurance companies [1]. Despite all this, the adherence rates stay low among eligible patients [1]. This is an interesting but worrisome conclusion since this is also the fact in other rehabilitation practices as well as in the newer preoperative rehabilitation (prehab) studies and settings [5-7].

In general, rehabilitation is well investigated and in chronic diseases, like chronic obstructive pulmonary disease (COPD), cardiovascular disease, neuromuscular disease, cancer, and even psychiatric disease (such as depression), it has been shown that exercise therapy, in particular, has beneficial effects on morbidity, mortality, and convalescence of these patients [5-9].

This review aims to give an overview of the barriers and facilitators in rehabilitation practices and possible reasons why adherence rates remain low.

## Review

Adherence in rehabilitation

According to the Agency for Healthcare Research and Quality (AHRQ), only 20% of the one million eligible patients (history of myocardial infarction, congestive heart failure, angioplasty, or heart surgery) participate in cardiac rehabilitation [1,3]. Cardiac rehabilitation is seen as the standard way of care after the abovementioned events [1,3]. The adherence rates are even lowering among females, ethnic or racial minorities, and in patients with low-income [1,3].

Even though the evidence for cardiac rehabilitation is clear, there is still not enough familiarity between the cardiologist and other specialties (in charge of) referring the patients [1]. Not every medical specialist is familiar with the mandatory amount of rehabilitation sessions that is advised by guidelines [1]. There are clear differences in referral rates between hospitals. Hospitals that made the referral process easier for their physicians have higher referral rates among eligible patients [1]. Similar problems were seen in prescribing supervised exercise therapy for patients with peripheral arterial occlusive disease (PAOD) in the Netherlands. After implementing ClaudicatioNet (now a part of ChronicCareNet), these problems significantly improved, resulting in a significantly higher referral rate [10-13]. If cardiac rehabilitation participation in the eligible group of patients would increase to 70%, there would be an enormous increase in saved lives (25,000). There would be 180,000 fewer patients who need to be hospitalized [14].

Another major procedure that could benefit from exercise pre- or post-surgery is bariatric surgery [7]. The patients who exercise before or after surgery have a lower risk of a cardiovascular event and experience a positive effect on physical fitness and anthropometrics [7]. The adherence levels in this field are relatively higher than in the abovementioned cardiac rehabilitation. Pouwels et al. showed in their systematic review that adherence levels in non-supervised and supervised exercise had a median of 57.3% (32.5-77.6%) and 64.5% (47.5-79.9%), respectively, which was seen as satisfactory by the researchers. No particular reasons were given for this specific adherence rate [7].

Looking at the complete group of patients that are recommended to have any form of rehabilitation, some studies estimate non-adherence levels up to 50% [15]. As a result, non-compliance could lead to physicians having to change their treatment plan unnecessarily, referring patients excessively, and, ultimately, still facing patients with unresolved long-term health complaints. Overall, non-adherence could result in a greater economic burden on the healthcare system [15-17].

Barriers

Several barriers could cause these aforementioned low adherence rates. One of these barriers could be that not all the treating physicians refer the patients properly or when necessary. It is postulated that this could be due to a lack of study guideline updates and dissemination [1,13]. This could cause a delay or complete lack of participation in these programs [1,13].

Another possible barrier could be that the referral process is more time-consuming and difficult in certain hospitals, causing a higher threshold for physicians to refer the patients to the proper rehabilitation program. Distance is also a contributing factor to low adherence rates. Some programs are located at specific places that are not easy to attain for everybody [2].

In the systematic review performed by Jack et al. regarding barriers to treatment adherence in physiotherapy outpatient clinics, several reasons/barriers were found [2]. The most important factors and/or barriers that lead to low adherence levels were low levels of physical activity at the beginning of the programs, low self-efficacy, depression, greater (subjective) perceived number of barriers, high levels of pain during exercise, anxiety, helplessness, and poor social support system [2]. Jack et al. mentioned that patients with a poor social support system have lower adherence [2]. Maybe this also leads back to the fact that this group of patients could be part of a group with a lower socioeconomic status, for which it is hard to pay the possible (part of the) costs. This study was not conclusive on whether or not age and greater pain at baseline were relevant contributors to lower adherence rates [2]. The rationale was that younger patients without an athletic identity and older patients without a social support network, among other things, would have lower adherence rates [2].

Possibly one of the most important barriers is the lack of motivation among the eligible patients. Without the proper motivation, it could be hard to start rehabilitation therapy and even more difficult to complete the entire program. Also, a good explanation of the program is essential for the patients' adherence [2,4,18]. Some of these barriers could and need to be addressed in treatment plans. For example, pain during exercise can be diminished or even avoided by sufficient analgesics or other interventions [2,4,18].

Facilitators

Besides the potentially long list of barriers, there are also some facilitators for patients undergoing any form of rehabilitation. In the abovementioned part, we discussed that low self-efficacy could harm adherence to a rehabilitation program. High self-efficacy has been linked to higher adherence rates in both orthopedic and musculoskeletal cohorts [15]. A study investigating adherence to sports injury rehabilitation programs indicates no correlation between self-efficacy and (sports) rehabilitation. However, this study was performed in a very specific cohort and possibly not applicable to less specific cohorts [19].

The way in which patients perceive their impairment affects their adherence. The three pillars that play a role in this process are 1) their own beliefs, 2) personal experience, and 3) received information [20]. Taylor et al. stated that the more serious a condition is perceived by the patient, the higher the adherence [20]. A discussion exists on the effect of the information load because some studies suggest that providing more information could be beneficial, while other studies suggest that overloading the patients with information could create more confusion [15,21].

The levels of bodily and/or extremity pain during or at the start of the program are not clearly facilitators because there is no consensus about pain's effect on the adherence level [15]. More factors could often contribute to the adherence to the pain, for example, type of personality [15]. Other factors that could contribute to the facilitation of better adherence rates are, among others, better physical activity at baseline, patients without any mental illnesses, and patients with a good social support system [15].

Another newer facilitator could be in the form of a coach. This is one of the innovations that could have a positive effect on adherence rates. It is part of the connected health solutions [15]. By incorporating regularly combined feedback, supervision, and reinforcement strategies in the patient's home environment, there is more coaching input than in the clinical setting [15]. This form of coaching is possible by several online cloud-based portals, which have many services such as video calling, instant messaging, or e-mail [15]. This method has already been proven to be a good facilitator for physiotherapists and has led to higher adherence rates [15,21]. In the field of telemedicine, many innovative concepts have been introduced. Table 1 gives an overview of the differences between Electronic Health (E-Health) and Mobile Health (M-Health). Telerehabilitation (by video-conferencing) had already been investigated by Tousignant et al., and they found that this is as effective as usual care while potentially increasing access to services [22].

**Table 1:** Overview in difference between in E-Health and M-Health E-Health: electronic heath; M-Health: mobile health

Telemedicine	
E-Health	M-Health
Telemonitoring	Self management
Tele health visit	Self monitoring
Tele consultation	Patient education
Tele conferencing	Patient support
	Patient Reported Outcome (PRO) collection

Perioperative surgical rehabilitation

As defined by the WHO, rehabilitation includes a number of actions to help people experiencing or exposed to a disability to achieve and maintain optimal functioning in interaction with their environment.

Due to changes related to the aging of the human body, the increase in the incidence of chronic diseases, and consequently, progressive disability, older adults are the largest group of rehabilitation services beneficiaries. Surgery poses a major challenge for the senior organism and always poses a threat to maintaining the performance of the older patient. Age per se, as a rule, is no longer a contraindication to surgical intervention but is associated with a higher risk of perioperative complications. The goal of prehab is multimodal; 1) preparing the patient for surgery relief of adverse consequences, 2) prevention and therapy of complications after surgical treatment restoration, and 3) improvement of functioning after surgery.

Surgery, despite the undoubted benefits of this type of therapy, is a heavy burden for every patient, especially for the senior organism, and poses a significant threat to functional performance maintenance. In urgent procedures, it is usually impossible to apply the patient's preparation program for the procedure. However, when the procedure is carried out on a scheduled procedure, pre-preparing the patient and planning cooperation in the postoperative period can bring tangible benefits.

Preoperative assessment of older patients allows the prediction of potential complications [23]. Implementation of the rehabilitation program is especially important in patients with frailty syndrome and sarcopenia [24]. The occurrence of these syndromes is associated with a higher risk of intraoperative and postoperative complications, higher mortality, longer hospitalization after surgery, and higher care costs. In 2012 it was shown that the frailty index was a stronger predictor of mortality than age in almost 30,000 Europeans aged above 50 years [25].

There are reports that programs conducted before surgery (three to eight weeks) primarily based on exercise and dietary interventions increase patients' functional reserves and improve the operative results and reduce the risk of complications. Such programs shall include: 1) Health optimization - control of concomitant diseases, quitting addictions, control, and modification of medications taken; 2) Interventions based on physical activity/exercise. The program should include both aerobic and resistance exercises, preferably with the supervision of an exercise physiologist and/or physiotherapist; 3) Nutritional interventions related to the replenishment of nutritional deficiencies and optimization of nutrient reserves before surgery to compensate for catabolic processes after surgery and prevent loss of lean body mass. This includes micronutrients and adequate protein intake; 4) Lowering anxiety and mood disorders.

A common phenomenon in patients with frailty syndrome is postoperative delirium. Research indicates that cognitive training used before surgery can reduce this complication, so in some of the rehabilitation programs described in the literature, this type of intervention is also used [23,25-27].

**Figure 1:**
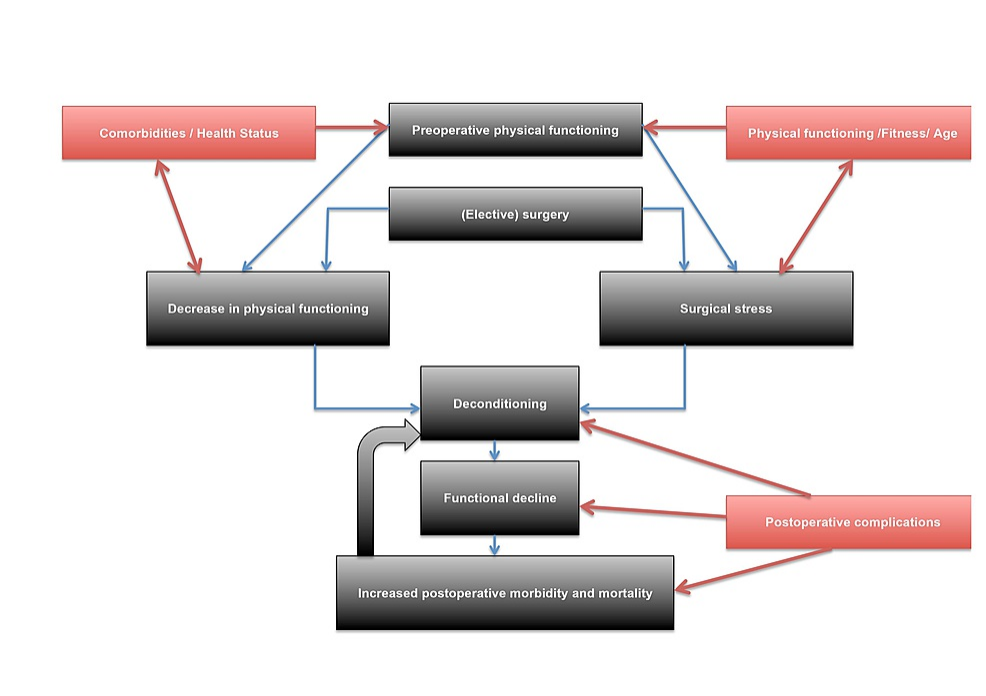
Overview of the interaction between physical functioning, preoperative health status, surgical stress, and postoperative morbidity and complications

After surgery, the primary purpose of rehabilitation is to return the patient to self-functioning in the environment as soon as possible. The patient's situation before the procedure, in many cases, leads to a decrease in motor activity and, consequently, to a decrease in physical performance and muscle strength. Surgery further exacerbates this problem. Figure 1 gives an overview of the complex interaction between physical functioning, preoperative health status, surgical stress, and postoperative morbidity and complications.

In the last years, a new rehabilitation field has emerged, the so-called preoperative rehabilitation (prehab) or preoperative exercise therapy (PET) in patients scheduled for surgery [5-8]. Several studies have investigated that the preoperative physical function is an independent predictor of postoperative morbidity and mortality in major abdominal cardiac and thoracic surgery [8]. Surgical stress leads to a decrease in physical functioning through different pathways; prolonged periods of physical inactivity also increase morbidity and mortality in the postoperative period. Finally, all of these phenomena will result in a decreased quality of life [28]. Preoperative physical capacity is a predictor of postoperative recovery, especially in elderly patients. Therefore we need to start the rehabilitation preoperatively, indicated by a significant evidence body [5-8,29]. PET aims to reduce the complication rate, length of hospital stay, and time of convalescence [5-8,29]. However, based on several reviews, univocal conclusions cannot be obtained in terms that PET can significantly reduce postoperative complications. Interestingly, PET has shown beneficial effects but, like in cardiac rehabilitation, as stated in the article by Rubin et al. [1], adherence levels remain low. Multiple barriers might influence adherence levels in these cases; 1) service and system-level barriers (physician recommendations and misconceptions about the rehabilitation program), 2) practical barriers (transport and parking), and 3) personal barriers (perceptions of the ability to control the disease) [1,5-8,29].

Regarding PET, there are also problems in the current body of literature, which were assessed in detail in the study by Pouwels et al. [8]. The majority of the studies are difficult to compare due to heterogeneous patient populations, non-comparable PET programs, and lack of guidelines for the use of PET programs and reported outcome measures. A fourth problem is the timing of PET around surgery, which can also influence the adherence level and, finally, the PET program's results [8,29,30].

Especially in oncological patients, there is a small preoperative time window (from diagnosis to surgery), which can influence the PET program's intensity. Also, these particular patients are often more difficult to train. This is because of the systemic effects of cancer and possibly chemo and/or radiotherapy. This often results in increased fatigue and less trainability, which will eventually decrease adherence rates. A shorter exercise program with a higher frequency of exercise sessions can be a problem for relatively unfit patients [6]. So far, the majority of the studies investigating PET use the physical activity guidelines for adults, which recommends exercise sessions of 30 minutes five times a week [1,5-8,29,30].

All in all, PET, a relatively new entity in rehabilitation, could potentially benefit postoperative recovery. However, this form of rehabilitation has certain system-specific, practical, and patient-specific barriers to overcome.

## Conclusions

We aimed to outline the barriers and facilitators for rehabilitation programs in chronic disease or postoperatively. Almost all the studies showed that these rehabilitation programs could positively affect patient outcomes, but the adherence rates are still too low. To improve the adherence rates, it is necessary to look at these barriers and try to dissolve them. Regarding potential future research, barriers and facilitators also need to be taken into account. Despite promising research in the field of PET and prehab and the enormous body of evidence in postoperative rehabilitation or rehabilitation in chronic diseases, we also need to take into account the accessibility of these prehab facilities in research and in clinical practice. Both barriers and facilitators need to be included in future research addressing prehab in clinical practice. 
